# A geo-coded inventory of anophelines in the Afrotropical Region south of the Sahara: 1898-2016

**DOI:** 10.12688/wellcomeopenres.12187.1

**Published:** 2017-07-26

**Authors:** David Kyalo, Punam Amratia, Clara W. Mundia, Charles M. Mbogo, Maureen Coetzee, Robert W. Snow

**Affiliations:** 1 Kenya Medical Research Institute-Wellcome Trust Research Programme, Nairobi, Kenya; 2 Centre for Emerging, Zoonotic & Parasitic Diseases, National Institute for Communicable Diseases, Johannesburg, South Africa; 3 Wits Research Institute for Malaria, School of Pathology, Faculty of Health Sciences, University of the Witwatersrand, Johannesburg, South Africa; 4 Centre for Tropical Medicine and Global Health, Nuffield Department of Clinical Medicine, University of Oxford, Oxford, UK

**Keywords:** Anopheles, Africa, Malaria, Vectors, Maps

## Abstract

**Background**: Understanding the distribution of anopheline vectors of malaria is an important prelude to the design of national malaria control and elimination programmes. A single, geo-coded continental inventory of anophelines using all available published and unpublished data has not been undertaken since the 1960s.

**Methods**: We have searched African, European and World Health Organization archives to identify unpublished reports on anopheline surveys in 48 sub-Saharan Africa countries. This search was supplemented by identification of reports that formed part of post-graduate theses, conference abstracts, regional insecticide resistance databases and more traditional bibliographic searches of peer-reviewed literature. Finally, a check was made against two recent repositories of dominant malaria vector species locations ( *circa* 2,500). Each report was used to extract information on the survey dates, village locations (geo-coded to provide a longitude and latitude), sampling methods, species identification methods and all anopheline species found present during the survey. Survey records were collapsed to a single site over time.

**Results**: The search strategy took years and resulted in 13,331 unique, geo-coded survey locations of anopheline vector occurrence between 1898 and 2016. A total of 12,204 (92%) sites reported the presence of 10 dominant vector species/sibling species; 4,473 (37%) of these sites were sampled since 2005. 4,442 (33%) sites reported at least one of 13 possible secondary vector species; 1,107 (25%) of these sites were sampled since 2005. Distributions of dominant and secondary vectors conform to previous descriptions of the ecological ranges of these vectors.

**Conclusion**: We have assembled the largest ever geo-coded database of anophelines in Africa, representing a legacy dataset for future updating and identification of knowledge gaps at national levels. The geo-coded database is available on Harvard Dataverse as a reference source for African national malaria control programmes planning their future control and elimination strategies.

## Introduction

In 1939, Botha de Meillon stated that “ *Malaria in South Africa, as elsewhere in the world, is an entomological disease. Its epidemiology only becomes clear when knowledge of its entomology has been elucidated*” ( De Meillon & Gear, 1939). This sentiment has been variously accepted and neglected throughout the history of malaria control in Africa.

The first global inventory of the Genus *Anopheles* (Diptera: Culicidae) was published in 1901 and reproduced in 1903 and 1910 ( Theobald, 1901). Sir Rickard Christophers updated this inventory in 1924 “ *as a necessary preliminary to studying the geographical distribution of species, has been published in the belief that, as a handy means of reference to known species with their correct names, it would be useful to medical men and others*” ( Christophers, 1924). In 1929, an assembly of reported locations of vectors from published and unpublished sources from the beginning of the 1900s was developed and presented as lists per country, which included location names and for the first time was shown on regional maps ( Kumm, 1929). This was updated for the Africa region in 1938, providing bibliographic sources, locations, taxonomic keys for adult mosquito stages and more details on bionomics by the Natural History Museum, London ( Evans, 1938), and repeated for larval stages in 1952 ( Hopkins, 1952). During the Second World War, the US Sanitary Department developed a separate inventory ( Ross & Roberts, 1943). 

The most definitive catalogue of recorded anopheline species for the Afrotropical region was published in 1968 by Mick Gillies and Botha de Meillon, updating the earlier work ( De Meillon, 1947) and capturing a wealth of published and unpublished observations from across the continent, linked to spatial grids of their distributions ( Gillies & de Meillon, 1968). This geo-referenced catalogue was accompanied by comprehensive descriptions of the morphology, notes on the species role in malaria transmission, and bionomics ( Gillies & de Meillon, 1968). Updated inventories of anopheline distributions were published in 1972 ( Fritz, 1972) and 1987 ( Gillies & Coetzee, 1987). More localised inventories were published on the distribution of the *Anopheles gambiae* complex in southern Africa ( Coetzee *et al.*, 1993) and on insecticide resistance in southern African vectors ( Coetzee *et al.*, 1999).

During the Global Malaria Eradication Programme (GMEP) era, from 1955–69, descriptions of the anopheline vectors, shown as sub-national distributions, were regarded as important preludes to pre-eradication and attack phases of eradication. The surveillance of malaria vectors in sub-Saharan Africa (SSA) was limited to those countries where eradication was pursued (e.g. the Horn of Africa, southern Africa and Africa’s offshore island states). When the emphasis on malaria control shifted to presumptive treatment of fevers through primary health care, entomological reconnaissance for malaria control became a forgotten public health science during the 1970s and 1980s across much of SSA ( Mnzava *et al.*, 2014).

In 1996, the Mapping Malaria Risk in Africa (MARA) collaboration was launched ( Coetzee *et al.*, 2000; Le Sueur *et al.*, 1997; Snow *et al.*, 1996; http://www.mara-database.org/) to assemble, geo-code and map malaria parasite and vector surveys undertaken across Africa, south of the Sahara. The initiative focused only on documenting records of the sibling species of the *An. gambiae* complex and *An. funestus s.l.* malaria vectors from reports of surveys undertaken between 1920 and 2004. This was a milestone collaboration, managed by scientists across the Africa region, and started a renaissance in the assembly of empirical malaria information as geo-coded inventories. Other initiatives to create entomological and insecticide resistance data repositories followed, including the African Network for Vector Resistance (ANVR) ( ANVR, 2005), the Malaria Atlas Project (MAP) ( Sinka *et al.*, 2010; http://www.map.ox.ac.uk; Wiebe *et al.*, 2017), the Disease Vectors Database ( Moffett *et al.*, 2009), MosquitoMap [ http://www.mosquitomap.org), Walter Reed Bioinformatics Unit’s systematic catalogue of Culicidae [ http://www.vectormap.org; Foley *et al.*, 2008; Foley *et al.*, 2010), IRBase [ http://www.irmapper.com; Dialynas *et al.*, 2009; Knox *et al.*, 2014), VectorBase [ http://www.vectorbase.org], which focuses on descriptions of bionomics and gene libraries of many disease vectors, and the Vector-Borne Disease Network (VecNet) [ http://www.vecnet.org].

The more recent on-line, global or regional, vector species location databases do not represent the entire historical reference material for any given country; they often focus only on peer-reviewed published sources without reference to unpublished reports from national control agencies and research partners, and they do not always cover the entire range of potential secondary vectors reported in countries. Here we present an assembly of geo-coded anopheline species data abstracted from survey reports published since 1900 across SSA and its offshore islands that have yet to eliminate malaria.

## Methods

### Data search

Methods used by us to identify sources of information have been opportunistic, cascaded approaches and began with bibliographies provided in earlier regional inventories published between 1929 – 1987 ( Evans, 1938; Fritz, 1972; Gillies & de Meillon, 1968; Gillies & Coetzee, 1987; Hopkins, 1952; Kumm, 1929). Manual searches were undertaken at the archives and libraries of ex-colonial tropical medicine institutes to locate unpublished reports from malariologists working in Africa before countries achieved independence. We searched the archives of the World Health Organization (WHO) in Geneva and regional archives in Brazzaville and Cairo, and identified consultant’s trip reports and quarterly reports from malariologists working on behalf of the WHO from the 1950s through to the 1970s. National archives of the Ministry of Health offices were visited in Burkina Faso, Ghana, Kenya, Senegal, South Africa, Sudan, Tanzania and Uganda. Annual medical and sanitation department reports from 1919, produced mostly by the Anglophone pre-independent colonial governments, were available at the library in the National Public Health Laboratories of the Ministry of Health, Nairobi, Kenya.

Post-graduate theses undertaken with entomological components were sourced from local university libraries in the faculties of zoology, medicine or related biological sciences in Kenya, Mali, Mozambique, Senegal, Sudan, Tanzania, Belgium, France and the UK. National and international malaria congresses and conference proceedings were also reviewed for abstracts that contained information on species identifications at specific localities. These sources of possible information are incomplete, and it is therefore to be expected that substantial data are available across university departments in Africa, not captured by us. Entomologists working across Africa, within research institutes or as part of National Malaria Control programmes, were also contacted directly for any unpublished survey reports, notably as part of more recent malaria vector surveillance and insecticide monitoring since the re-launch of indoor residual house-spraying in Africa.

Using more traditional methods of online electronic peer-review, published reports were searched using free text keywords " *Anopheles*" and " *country-name*" in March 2010 and repeated at least once per year before the search ended on 31 ^st^ March 2017. Bibliographic on-line resources included PubMed, Google Scholar and the World Health Organization Library Database. Regional journals, including a large number of national medical, public health and parasitological journals, were not identified readily from the above sources, but titles and abstracts were available on African Journals Online ( http://www.ajol.info). All publications were cross-referenced using the bibliographies for additional sources that may have been missed or that may correspond to unpublished or ‘grey’ literature, not controlled by commercial publishers. Finally, our database was compared with the 2,535 site location data reported by MARA/ARMA ( Coetzee *et al.*, 2000) and the 2,582 site locations recorded by MAP ( Wiebe *et al.*, 2017) to ensure these were captured as part of our search strategy. 

### Data abstraction

***Data organisation:*** The basic principle of the database was to develop a site-specific inventory. As such, multiple reports from the same site were collapsed to a single entry, with all citations combined to that site. Invariably, multiple authors of published material report on the same surveys or aspects of entomological work from the same site across a period of several years, and in these instances the first and last survey years were retained. Some bibliographic sources cite previous vector descriptions from unpublished sources by other entomologists; these have been documented as *op cit* to the original author against the reference source. For older reports it was not always possible to define the year of sampling, and where dates of sampling were not provided we have presumed that it happened within the preceding five years of the publication date. Separate reports on the same location vary in the stages of vector sampled and the precision methods used to distinguish species and sibling species of complexes. In such cases, all sampling, vector stage and species identification methods were recorded across surveys, often increasing with time in species identification precision.

***Sampling methods:*** For each record, we documented whether adults or larvae were sampled and a summary of methods used to sample vectors, for example animal bait catches, bed net traps, CDC light traps, human landing catches, human bait catches (where someone was protected by a double net), indoor resting searches, pyrethrum spray catches, exit traps, outdoor bait traps, Ifakara tent traps, Monks Wood traps, larval searches or larvae reared to adults. If there were no details available then "unknown" was recorded, which was often the case from national reviews of previously unpublished data. Insecticide resistance data often sample larvae at sites that were then reared to adults. In older literature, notably assemblies of unpublished data, it was not always possible to define whether adults or larvae were sampled and here we have defaulted to assuming both.

***Species identification:*** Throughout the data assembly we have only recorded the reported presence of a species where this was described during a survey. Even if the report stated absence of sibling species/species we have not recorded absence. True absence is not possible to define within an ecological niche of vector species, as it depends critically on the intensity, duration and repeat sampling at any given site.

### Dominant and potential secondary vectors

A perennial problem with assemblies of vector inventories over time are the ambiguities in taxonomy and nomenclature. These improve with time as part of detailed mosquito systematics, improvements in morphological keys and genetic techniques. Methods used to identify species were recorded for all surveys at each location as per morphological keys, cross-mating, Polymerase Chain Reaction (PCR), chromosome banding sequences, DNA probes or enzyme electrophoresis.

We have regarded as primary, dominant vectors within their ecological range as *An. gambiae s.l.* ( *An. gambiae s.s, An. coluzzii, An. arabiensis, An. melas, An. merus, An. bwambae*); *An. funestus s.s.; An. nili s.l.,* ( *An. nili s.s., An. carnevalei, An. ovengensis), An. moucheti s.l.* ( *An. moucheti moucheti, An. moucheti nigeriensis);* and *An. mascarensis* as a primary vector in Madagascar, Comoros and Mayotte ( Fontenille & Campbell, 1992; Fontenille *et al.*, 2003; Marrama *et al.*, 1999).

For the Gambiae complex, as much detail as possible from the reports was extracted and updated with new more specific chromosomal or cross-mating information from subsequent reports at the same location: *An. gambiae s.l.* (if only complex mentioned or *An. costalis* in very early reports), *An. gambiae s.s.* (Species A) when possible to differentiate from *An. arabiensis* (Species B) and saltwater breeding species *(An. melas* and *An. merus), An. gambiae* S form (when indicated as Savannah or Bamako or S forms) and *An. coluzzii* (when indicated as M form or Mopti form). The zoophilic *An. quadriannulatus* A and *An. quadriannulatus* B were described as sibling-species of the *An. gambiae* complex (previously species C) in the early 1980s, but not regarded as vectors of malaria within their geographic ranges of southern Africa and Ethiopia ( Coetzee, 1987; Coluzzi, 1984). *An. quadriannulatus* B from Ethiopia was later renamed *An. amharicus* Hunt, Wilkerson & Coetzee sp. n. ( Coetzee *et al.*, 2013; Hunt *et al.*, 1998) while the name *An. quadriannulatus* was retained for the southern African species. *An. quadriannulatus* is recorded under other species. *An. bwambae* is a member of the Gambiae complex involved in malaria transmission only within a very restricted geographical range in Uganda ( Davidson & Hunt, 1973; White, 1985).

The *An. funestus* group has taxonomic complexity similar to that of the Gambiae complex. The taxonomic classification and systematics of the *An. funestus* group has resulted in a reclassification of the group with *An. funestus s.s.*, *An. aruni*, *An. parensis*, *An. confusus, An. vaneedeni* and *An. funestus-like* (described in Malawi ( Spillings *et al.*, 2009)) being grouped together as members of the “ *An. funestus* subgroup”; *An. rivulorum*, *An. rivulorum-like*, *An. brucei* and *An. fuscivenosus* form their own subgroup; and *An. leesoni* has been grouped with the Asian *An. minimus* subgroup ( Choi *et al.*, 2012; Coetzee & Koekemoer, 2013; Harbach, 2004; Spillings *et al.*, 2009). Among the Funestus group, *An. funestus s.s.* is a significant vector in the transmission of malaria ( Coetzee & Koekemoer, 2013); *An. rivulorum* has been recently implicated in transmission in Tanzania and might contribute as a secondary vector to transmission elsewhere ( Kawada *et al.*, 2012; Wilkes *et al.*, 1996); and *An. vaneedeni* was implicated recently in residual transmission in South Africa ( Burke *et al.*, 2017). We have documented only the presence of sibling species within groups where these have been uniquely defined in the reports.

*An. moucheti* is an important vector in equatorial forests in Central and West Africa ( Antonio-Nkondjio *et al.*, 2002; Antonio-Nkondjio *et al.*, 2008; Mattingly, 1949; Manga *et al.*, 1995). This vector was originally divided into three morphological forms *An. moucheti moucheti* (type form), *An. moucheti bervoetsi* and *An. moucheti nigeriensis* ( Brunhes *et al.*, 1998). However, recent classifications recognize *An. moucheti* and *An. bervoetsi* as formal species, while *An. moucheti nigeriensis* is considered as a morphological subspecies of *An. moucheti* ( Antonio-Nkondjio *et al.*, 2002; Antonio-Nkondjio *et al.*, 2008).

The *An. nili* complex currently comprises four formal species, *An. nili s.s.*, *An. somalicus*, *An. carnevalei* and *An. ovengensis* ( Awono-Ambene *et al.*, 2004; Kengne *et al.*, 2003) *. An. somalicus*, has never been incriminated in human malaria transmission, however the three other members are highly anthropophilic and are important vectors of malaria within most of their geographical range ( Carnevale & Zoulani, 1975; Mouchet *et al.*, 2008).

The definition of secondary vectors is complex and often site/time specific ( Afrane *et al.*, 2016; De Meillon, 1951; Gillies & de Meillon, 1968; Holstein, 1951). Most non-dominant, potential vectors are exophilic (outdoor resting), exophagic (outdoor biting) and zoophilic (preference, but not exclusive, for non-human hosts). They only feature as possible vectors of malaria where they are abundant and have life-expectancies long enough to support transmission. The single most important characteristic is whether the vectors have been detected harbouring *Plasmodium falciparum* sporozoites in their salivary glands, suggesting they have acquired infections from human hosts and have survived long enough for a complete sporogonic cycle. We have proposed a revised list of potential secondary vectors based on historical and contemporary reports of detection of sporozoite infected adult sampled species ( Table 1). Despite occasional reference to sporozoite positivity in several locations before 1950 ( De Meillon, 1950), we have not treated *An. austeni, An. brunnipes, An. christyi, An. hargreavesi, An. maculipalpis, An. theileri, An. pretoriensis* and *An. rhodesiensis as* secondary vectors; however they are documented within the database alongside other anopheline species detected at sites across Africa. In addition, we have not included *An. dthali* as a secondary vector, since reports of contributions to transmission in the arid areas of the Horn of Africa are extremely localized ( Lega *et al.*, 1937; Rishikesh, 1961). Records under the “other anophelines” field were listed per the taxonomic older synonym or variety names, but subsequently corrected to nomenclature used today.

**Table 1.** List of potential secondary vectors described as being sporozoite positive during field surveys in 20 countries since 1929.

Secondary malaria vectors	Location/country where sporozoite positive samples found	Citation
*Anopheles* *pharoensis*	Benin, Burkina Faso, Chad, Cameroon, Ethiopia, Ghana, Kenya, Mali, Nigeria, Senegal, Tanzania, Uganda	Animut *et al.*, 2013; Antonio-Nkondjio *et al.*, 2006; Carrara *et al.*, 1990; Cavalie & Mouchet, 1961; De Meillon, 1950; Dery *et al.*, 2010; Dia *et al.*, 2008; Gari *et al.*, 2016; Gibbins, 1932; Gillies, 1964; Hamon *et al.*, 1956; Kerah-Hinzoumbé *et al.*, 2009; Kibret *et al.*, 2014; Miles *et al.*, 1983; Mukiama & Mwangi, 1989; Tchouassi *et al.*, 2012; Wanji *et al.*, 2003
*Anopheles* *squamosus* ^1^	Kenya, Mali, Tanzania, Zambia	De Meillon, 1947; Fornadel *et al.*, 2011; Gillies, 1964; Hamon *et al.*, 1956; Lobo *et al.*, 2015; St Laurent *et al.*, 2016
*Anopheles* *wellcomei*	Cameroon, Senegal	Antonio-Nkondjio *et al.*, 2006; Dia *et al.*, 2008; Wanji *et al.*, 2003
*Anopheles* *rufipes*	Burkina Faso, Cameroon, Gambia, Ghana, Kenya, Mali, Nigeria, Senegal, Togo	Da *et al.*, 2013; Dery *et al.*, 2010; Dia *et al.*, 2008; Gelfand, 1947; Hamon & Rickenbach, 1955; Hamon *et al.*, 1956; Holstein, 1960; St Laurent *et al.*, 2016; Tabue *et al.*, 2017; Tchouassi *et al.*, 2012
*Anopheles* *hancocki*	Cameroon, Uganda	Antonio-Nkondjio *et al.*, 2006; De Meillon, 1950; Gibbins, 1932; Wanji *et al.*, 2003
*Anopheles* *marshallii* ^2^	Cameroon, Uganda	Antonio-Nkondjio *et al.*, 2006; Bafort, 1985; De Meillon, 1950; Gibbins, 1932; Wanji *et al.*, 2003
**Funestus subgroup**		
*Anopheles* *leesoni*	Kenya, South Africa, Tanzania	Conn, 2016; Garros *et al.*, 2004; St Laurent *et al.*, 2016
*Anopheles* *parensis*	Kenya, South Africa	Garros *et al.*, 2004; Kamau *et al.*, 2003
*Anopheles* *vaneedeni*	South Africa	Burke *et al.*, 2017; Garros *et al.*, 2004; Green & Hunt, 1980
*Anopheles* *rivulorum*	Kenya, Tanzania, Zambia	Gillies, 1964; Lobo *et al.*, 2015; Wilkes *et al.*, 1996
**Coustani group** ^3^		
*Anopheles* *coustani*	Benin, Burkina Faso, Cameroon, DRC, Ethiopia, Ghana, Kenya, Madagascar, Mozambique, Tanzania, Zambia	Antonio-Nkondjio *et al.*, 2006; Fornadel *et al.*, 2011; Gillies, 1964; Govoetchan *et al.*, 2014; Hamon & Mouchet, 1961; Kibret *et al.*, 2014; Lobo *et al.*, 2015; Mendis *et al.*, 2000; Mwangangi *et al.*, 2013; Nepomichene *et al.*, 2015; Sovi *et al.*, 2013; Tchouassi *et al.*, 2012; Vincke & Jadin, 1946
*Anopheles* *paludis*	Cameroon, DRC	Antonio-Nkondjio *et al.*, 2006; De Meillon, 1950; Karch & Mouchet, 1992; Lips, 1961; Wanji *et al.*, 2003
*Anopheles* *ziemanni*	Benin, Burkina Faso, Cameroon, Chad, Cote d'Ivoire, Ethiopia, Kenya, Tanzania	Animut *et al.*, 2013; Gari *et al.*, 2016; Githeko *et al.*, 1990; Gillies & de Meillon, 1968; Govoetchan *et al.*, 2014; Hamon & Mouchet, 1961; Kamau *et al.*, 2006; Kerah-Hinzoumbé *et al.*, 2009; Lobo *et al.*, 2015; Robert *et al.*, 1992; Sovi *et al.*, 2013; Vincke & Jadin, 1946; Wilkes *et al.*, 1996

**Notes** ^1^ *An. squamosus* in Madagascar was variously recorded as *An. squamous*, *An. squamosus* var. *cydippis* [ Nepomichene *et al.*, 2015] and later unique reports of *An. cydippis*. The taxonomical keys reported in individual reports are vague and we have recorded *An. squamous* presence where used in the species report, but as *An. cydippis* under other species where specified without reference to *An. squamosus*. ^2^ *An. marshallii* is a complex, although rarely differentiated during field surveys except when specified as *An. keniensis*. In Madagascar, early surveys often mis-classified *An. mascarensis* as *An. marshallii*. ^3^The taxonomic name of *An. mauritianus* was sunk as a synonym of *An. coustani* in 1932. The varieties of *tenebrosus* and *ziemanni* were raised to species status by Gillies & de Meillon (1968), while *An. paludis* was a recognised species by Evans (1938). Where early reports document *An. mauritianus* and the varieties, the current species names have been recorded here.

### Survey geo-coding

Each survey location was attributed a decimal longitude and latitude. Where household level data were reported these were re-aggregated to village levels as a single entry. Where only mapped distributions of species locations were provided, without location names, these were overlaid in digital formats with Google Earth and location/site names extracted. Mosquito systematics, however, often reported longitude and latitude of survey locations and detailed descriptions of survey sites, and the more recent use of Global Positioning Systems (GPS) during survey work has increased reporting of longitudes and latitudes. All reported coordinates were re-checked in Google Earth. For geo-referencing survey locations, where longitudes and latitudes were not available in the original survey reports, we have used a variety of digital resources, amongst which the most useful were Microsoft Encarta Encyclopedia, Google Earth, GeoNames and OpenStreetData. Other sources of digital place name archives used are shown in Table 2 and used in combination with increasingly available national statistics bureau, ministry of health or ministry of education geo-coded place name databases.

**Table 2.** Other on-line digital placename gazetteers used during geo-coding process.

Name	Link
NGA GEOnet	http://geonames.nga.mil/gns/html/
Geoview country portal	http://geoview.info/
IslamicFinder	http://www.islamicfinder.org/prayerDetail.php
UN OCHA humanitarian	https://data.humdata.org/dataset?sort=metadata_modified+desc
Falling Rain Genomics’ Global Gazetteer	http://www.fallingrain.com/world/index.html
Alexandria Digital Library	http://www.alexandria.ucsb.edu
ILRI Geoportal	http://data.ilri.org/geoportal/catalog/main/home.page
ArcGIS online	http://www.arcgis.com/home/webmap/viewer.html?useExisting=1
African Data Sampler	http://gcmd.nasa.gov/records/GCMD_ADS_WRI.html
MapCarta	http://mapcarta.com/
Maplandia	http://www.maplandia.com/
Global geodatabase- cities	http://www.geodatasource.com/
VMAP0	http://earth-info.nga.mil/publications/vmap0.html
Bing Maps	https://www.bing.com/maps/
Here Maps	https://wego.here.com/
Old maps online	http://www.oldmapsonline.org/
Mapcruzin	http://www.mapcruzin.com/free-africa-arcgis-maps-shapefiles.htm
CIESIN	http://sedac.ciesin.columbia.edu/data/set/grump-v1-settlement-points
FreeGIS data	https://freegisdata.rtwilson.com/
ILRI Geoportal	http://data.ilri.org/geoportal/catalog/main/home.page

### Results

We identified 2,221 published and unpublished reports. The earliest survey report was of *An. costalis* (now known as *An. gambiae s.l.*) collected in Freetown and its environs in Sierra Leone in expeditions carried out by Christophers and Stephens in 1898 and 1899 ( Christophers & Stephens, 1900; Stephens & Christophers, 1900). Important sources of information have been national inventories developed by entomologists working in Africa, the earliest were those developed before the launch of the GMEP in Cape Verde, Democratic Republic of Congo (DRC), Eritrea, Ethiopia, Gabon, Kenya, Liberia, Mozambique, Nigeria, Rwanda and Burundi, South Africa, Sudan, Zambia and Zanzibar. During the preparation for the GMEP, countries often undertook national malaria reconnaissance surveys that included detailed descriptions of local ecologies and vector habitats, host infection studies and the presence of all anophelines by species and stages. After the Second World War, work of individual malariologists and entomologists began to be assembled into national inventories of anopheline distributions and in several instances mapped to provide a visual display of the recorded species distributions. These early national inventories, for the most part, were based on unpublished survey data from ministry of health reports spanning decades of entomological investigation. Some countries have recognised the importance of updating inventories of anopheline distributions, notably where regional compendia are incomplete for country purposes. Examples of these more contemporary national inventories were identified for Cote d’Ivoire, DRC, Kenya, Madagascar, Mali, Mauritania, Niger, Nigeria, Senegal, Somalia and Tanzania.

**Figure 1.**
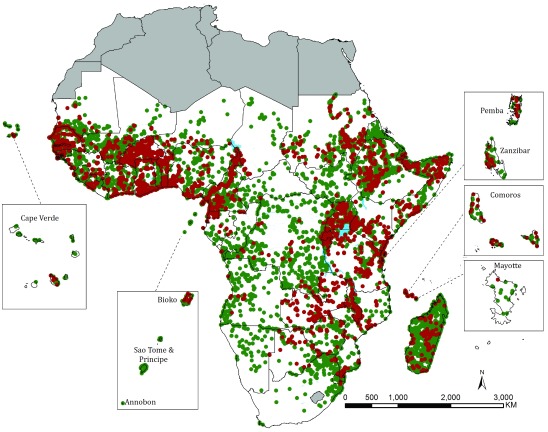
The spatial distribution of 13,331 Anopheline survey locations in Afrotropical Region south of the Sahara between 1898 and 2016. 4,494 sampling locations where survey dates included 2005–2016 shown in red.

The final database contained 13,465 unique survey locations where anophelines were sampled between 1898 and 2016. We were able to geo-locate 13,331 (99%) using a variety of on-line digital gazetteers ( Figure 1; Table 3). Sampled locations that have included surveys since 2005 cover 4,494 survey sites and highlight the paucity of available contemporary information on malaria vector species distributions in Congo, Central African Republic, Chad, Eritrea, Namibia, Sao Tome and Principe, South Sudan and Togo ( Figure 1; Table 3).

**Table 3.** Numbers of geo-coded site locations documenting dominant vector species (DVS) and potential secondary vector species (SVS) by country and lists of all anophelines identified during entomological surveys in the current database and supplemented by anopheline descriptions provided in [ Gillies & de Meillon, 1968; White, 1980].

Country	DVS survey locations (date range) [No. locations since 2005]	SVS survey locations 1900–2016 [No. locations since 2005]	Lists of anophelines described
**Angola**	**195** (1904–2014) [22]	**119** [12]	*Anopheles arabiensis, An. ardensis, An. argenteolobatus, An. austeni, An. azevedoi, An. barberellus,* *An. brunnipes, An. caliginosus, An. cinctus, An. coluzzii, An. concolor, An. coustani, An. cydippis,* *An. demeilloni, An. distinctus, An. dureni, An. flavicosta, An. funestus s.s., An. fuscivenosus,* *An. gambiae s.s., An. hancocki, An. harperi, An. implexus, An. jebudensis, An. leesoni, An. listeri,* *An. longipalpis, An. maculipalpis, An. marshallii, An. melas, An. natalensis, An. nili, An. njombiensis, An. obscurus, An. paludis,* *An. pharoensis, An. pretoriensis, An. rhodesiensis, An. rivulorum,* *An. ruarinus, An. rufipes, An. schwetzi, An. squamosus, An. tchekedii, An. tenebrosus, An. theileri, An. walravensi, An. wellcomei,* *An. wellcomei ugandae, An. ziemanni*
**Benin**	**399** (1905–2016) [320]	**138** [101]	*An. arabiensis, An. brohieri, An. brunnipes, An. coluzzii, An. coustani, An. domicolus, An. flavicosta, An. funestus s.s.,* *An. fuscicolor, An. gambiae s.s., An. hargreavesi, An. leesoni, An. maculipalpis,* *An. melas, An. nili, An. obscurus, An. paludis, An. pharoensis, An. pretoriensis, An. rhodesiensis,* *An. rivulorum, An. rufipes, An. smithii, An. squamosus, An. wellcomei, An. ziemanni*
**Botswana**	**82** (1961–2015) [34]	**33** [8]	*An. arabiensis, An. argentolobatus, An. caliginosus, An. coustani, An. cydippis, An. demeilloni,* *An. distinctus, An. funestus s.l., An. leesoni, An. listeri, An. longipalpis, An. maculipalpis,* *An. marshallii, An. parensis, An. pharoensis, An. pretoriensis, An. quadriannulatus, An. rhodesiensis, An. rivulorum, An. rufipes,* *An. seretsei, An. squamosus, An. tchekedii, An. tenebrosus, An. vaneedeni, An. walravensi, An. wellcomei ugandae, An. ziemanni*
**Burkina Faso**	**550** (1939–2014) [400]	**72** [38]	*An. arabiensis, An argenteolobatus, An. brohieri, An. brunnipes, An. coluzzii, An. coustani,* *An. domicolus, An. flavicosta, An. funestus s.s., An. gambiae s.s., An. hancocki, An. leesoni,* *An. longipalpis, An. maculipalpis, An. moucheti, An. natalensis, An. nili, An. pharoensis,* *An. pretoriensis, An. rivulorum, An. rufipes, An. sergentii, An. squamosus, An. subpictus,* *An. wellcomei, An. ziemanni*
**Burundi**	**57** (1935–2014) [15]	**49** [1]	*An. arabiensis, An. ardensis, An. christyi, An. coustani, An. cydippis, An. demeilloni, An. funestus s.s.,* *An. gambiae s.s., An. garnhami, An. gibbinsi, An. implexus, An. longipalpis, An. maculipalpis,* *An. marshallii, An. moucheti, An. natalensis, An. nili, An. pharoensis, An. seydeli, An. squamosus,* *An. theileri, An. wellcomei ugandae, An. ziemanni*
**Cameroon**	**879** (1907–2015) [540]	**220** [86]	*An. arabiensis, An. bervoetsi, An. brohieri, An. brunnipes, An. buxtoni, An. carnevalei, An. christyi, An. cinctus,* *An. coluzzii, An. concolor, An. coustani, An. cydippis, An. deeming, An. demeilloni,* *An. domicolus, An. dualaensis, An. eouzani, An. flavicosta, An. freetownensis, An. funestus s.s.,* *An. gambiae s.s., An. hancocki, An. hargreavesi, An. implexus, An. jebudensis, An. leesoni,* *An. longipalpis, An. maculipalpis, An. marshallii, An. melas, An. moucheti, An. mousinhoi,* *An. multicinctus, An. namibiensis, An. natalensis, An. nigeriensis, An. nili, An. obscurus,* *An. ovengensis, An. paludis, An. pharoensis, An. pretoriensis, An. rageaui, An. rhodesiensis,* *An. rivulorum, An. rufipes, An. sergentii, An. smithii, An. somalicus, An. squamosus, An. tenebrosus, An. theileri, An. wellcomei,* *An. ziemanni*
**Cape Verde**	**48** (1942–2011) [5]	**0**	*An. arabiensis, An. pretoriensis*
**Central** **African** **Republic**	**54** (1950–2014) [20]	**23** [5]	*An. brohieri, An. cinctus, An. coluzzii, An. coustani, An. cydippis, An. domicolus, An. flavicosta,* *An. freetownensis, An. funestus s.l., An. gambiae s.s., An. hancocki, An. hargreavesi, An. implexus,* *An. leesoni, An. longipalpis, An. maculipalpis, An. marshallii, An. moucheti, An. natalensis, An. nili, An. obscurus, An. paludis,* *An. pharoensis, An. pretoriensis, An. rhodesiensis, An. rufipes,* *An. squamosus, An. wellcomei, An. ziemanni*
**Chad**	**34** (1950–2014) [14]	**28** [5]	*An. arabiensis, An. christyi, An. cinctus, An. cinereus, An. coluzzii, An. coustani, An. cydippis,* *An. dthali, An. funestus s.s., An. gambiae s.s., An. nili, An. pharoensis, An. rhodesiensis, An. rufipes,* *An. sergentii, An. squamosus, An. wellcomei, An. ziemanni*
**Comoros**	**56** (1952–2011) [19]	**12** [2]	*An. arabiensis, An. coluzzii, An. comorensis, An. coustani, An. funestus s.s., An. gambiae s.s.,* *An. maculipalpis, An. mascarensis, An. merus, An. pretoriensis*
**Congo**	**73** (1943–2009) [2]	**37** [1]	*An. ardensis, An. barberellus, An. brohieri, An. brunnipes, An. caroni, An. cinctus, An. cinereus,* *An. coluzzii, An. coustani, An. cydippis, An. demeilloni, An. freetownensis, An. funestus s.l.,* *An. gambiae s.s., An. hamoni, An. hancocki, An. hargreavesi, An. implexus, An. jebudensis,* *An. leesoni, An. longipalpis, An. marshallii, An. melas, An. moucheti, An. nili, An. obscurus,* *An. paludis, An. pretoriensis, An. rhodesiensis, An. rivulorum, An. rufipes, An. squamosus,* *An. ziemanni*
**Cote d’Ivoire**	**325** (1902–2015) [104]	**83** [36]	*An. arabiensis, An. barberellus, An. brohieri, An. brunnipes, An. cinctus, An. coluzzii, An. coustani, An. demeilloni, An. domicolus,* *An. dureni, An. flavicosta, An. freetownensis, An. funestus s.s.,* *An. gambiae s.s., An. hancocki, An. hargreavesi, An. implexus, An. jebudensis, An. leesoni,* *An. maculipalpis, An. marshallii, An. melas, An. natalensis, An. nili, An. obscurus, An. paludis,* *An. pharoensis, An. pretoriensis, An. rhodesiensis, An. rivulorum, An. rodhaini, An. rufipes,* *An. smithii, An. squamosus, An. wellcomei, An. ziemanni*
**Democratic** **Republic of** **Congo**	**515** (1902–2014) [41]	**314** [18]	*An. arabiensis, An. ardensis, An. argenteolobatus, An. austeni, An. barberellus, An. berghei,* *An. bervoetsi, An. brunnipes, An. caliginosus, An. christyi, An. cinctus, An. coluzzii, An. concolor,* *An. confusus, An. coustani, An. cydippis, An. demeilloni, An. distinctus, An. domicolus, An. dureni,* *An. faini, An. funestus s.l., An. gambiae s.s., An. garnhami, An. gibbinsi, An. hancocki, An. hargreavesi,* *An. implexus, An. jebudensis, An. keniensis, An. kingi, An. leesoni, An. longipalpis, An. maculipalpis, An. marshallii, An. melas,* *An. mortiauxi, An. moucheti, An. mousinhoi, An. multicinctus,* *An. natalensis, An. nili, An. njombiensis, An. obscurus, An. paludis, An. pharoensis, An. pretoriensis, An. rhodesiensis,* *An. rivulorum, An. rodhaini, An. rufipes, An. schwetzi, An. seydeli, An. squamosus, An. symesi, An. tenebrosus, An. theileri,* *An. ugandae, An. vanhoofi, An. vinckei, An. walravensi,* *An. wellcomei, An. ziemanni*
**Djibouti**	**35** (1910–2011) [12]	**0**	*An. azaniae, An. dthali, An. dancalicus, An. gambiae s.l., An. harperi, An. rhodesiensis, An. salbaii,* *An. sergentii, An. sergentii macmahoni, An. turkhudi*
**Equatorial Guinea**	**92** (1933–2014) [49]	**0**	*An. brunnipes, An. carnevalei, An. cinctus, An. coluzzii, An. funestus s.l., An. gambiae s.s., An. lloreti,* *An. melas, An. moucheti, An. nili, An. obscurus, An. smithii*
**Eritrea**	**77** (1936–2003) [0]	**18** [0]	*An. arabiensis, An. christyi, An. cinereus, An. coustani, An. d'thali, An. dancalicus, An. demeilloni,* *An. erythraeus, An. funestus s.l., An. garnhami, An. nili, An. pharoensis, An. rhodesiensis,* *An. rhodesiensis rupicolus, An. rivulorum, An. rufipes, An. sergentii macmahoni, An. squamosus,* *An. turkhudi*
**Ethiopia**	**601** (1910–2015) [169]	**313** [83]	*An. amharicus, An. arabiensis, An. ardensis, An. christyi, An. cinereus, An. confusus, An. coustani, An. culicifacies (syn.* *adenensis), An. cydippis, An. dthali, An. dancalicus, An. demeilloni,* *An. domicolus, An. erythraeus, An. funestus s.s., An. garnhami, An. gibbinsi, An. harperi,* *An. implexus, An. kingi, An. leesoni, An. longipalpis, An. maculipalpis, An. marshallii, An. natalensis, An. nili, An. obscurus,* *An. paludis, An. parensis, An. pharoensis, An. pretoriensis, An. rhodesiensis,* *An. rhodesiensis rupicolus, An. rivulorum, An. rufipes, An. salbaii, An. sergentii macmahoni,* *An. seydeli, An. squamosus, An. tenebrosus, An. theileri, An. turkhudi, An. wellcomei, An. ziemanni*
**Gabon**	**35** (1930–2013) [13]	**23** [5]	*An. cinctus, An. coluzzii, An. coustani, An. faini, An. funestus s.l., An. gabonensis, An. gambiae s.s.,* *An. hancocki, An. hargreavesi, An. jebudensis, An. maculipalpis, An. marshallii, An. melas,* *An. moucheti, An. natalensis, An. nili, An. obscurus, An. paludis, An. pharoensis, An. pretoriensis,* *An. rhodesiensis, An. rufipes, An. schwetzi, An. smithii, An. tenebrosus, An. theileri, An. vinckei,* *An. wellcomei, An. ziemanni*
**Gambia**	**171** (1901–2014) [62]	**41** [18]	*An. arabiensis, An. brohieri, An. brunnipes, An. coluzzii, An. coustani, An. funestus s.l.,* *An. gambiae s.s., An. flavicosta, An. funestus, An. maculipalpis, An. melas, An. murphyi,* *An. nili, An. pharoensis, An. rufipes, An. squamosus, An. wellcomei, An. ziemanni*
**Ghana**	**409** (1900–2015) [278]	**80** [41]	*An. arabiensis, An. brohieri, An. brunnipes, An. cinctus, An. coluzzii, An. coustani, An. demeilloni,* *An. domicolus, An. flavicosta, An. freetownensis, An. funestus s.s., An. gambiae s.s., An. hancocki,* *An. hargreavesi, An. implexus, An. leesoni, An. maculipalpis, An. marshallii, An. melas, An. nili,* *An. obscurus, An. paludis, An. pharoensis, An. pretoriensis, An. rhodesiensis, An. rufipes, An. smithii, An. squamosus, An. theileri,* *An. watsoni, An. wellcomei, An. ziemannii*
**Guinea**	**92** (1903–2015) [38]	**34** [3]	*An. arabiensis, An. barberellus, An. brohieri, An. brunnipes, An. cavernicolus, An. cinctus,* *An. coluzzii, An. coustani, An. domicolus, An. flavicosta, An. freetownensis, An. funestus s.s.,* *An. gambiae s.s., An. hancocki, An. hargreavesi, An. implexus, An. leesoni, An. longipalpis,* *An. maculipalpis, An. maliensis, An. marshallii, An. melas, An. moucheti, An. nigeriensis, An. nili,* *An. obscurus, An. pharoensis, An. pretoriensis, An. rageaui, An. rhodesiensis, An. rivulorum,* *An. rufipes, An. sergentii, An. sergentii macmahoni, An. smithii, An. somalicus, An. squamosus,* *An. wellcomei, An. ziemanni*
**Guinea** **Bissau**	**56** (1927–2010) [17]	**4** [2]	*An. arabiensis, An. cinereus, An. coluzzii, An. coustani, An. dancalicus, An. funestus s.l.,* *An. gambiae s.s., An. hargreavesi, An. maculipalpis, An. melas, An. nili, An. pharoensis, An. rufipes, An. smithii, An. squamosus,* *An. ziemanni*
**Kenya**	**991** (1900–2015) [440]	**282** [144]	*An. arabiensis, An. ardensis, An. azaniae, An. christyi, An. cinereus, An. confusus, An. coustani,* *An. dthali, An. demeilloni, An. flavicosta, An. funestus s.s., An. gambiae s.s., An. garnhami,* *An. gibbinsi, An. harperi, An. implexus, An. keniensis, An. kingi, An. leesoni, An. longipalpis,* *An. lounibosi, An. maculipalpis, An. marshallii, An. merus, An. moucheti, An. multicinctus,* *An. natalensis, An. nili, An. paludis, An. parensis, An. pharoensis, An. pretoriensis, An. rabaiensis,* *An. rhodesiensis, An. rivulorum, An. rufipes, An. salbaii, An. sergentii, An. smithii, An. squamosus, An. swahilicus, An. symesi,* *An. tenebrosus, An. theileri, An. vaneedeni, An. wilsoni,* *An. wellcomei erepens, An. ziemanni*
**Liberia**	**246** (1902–2014) [39]	**91** [6]	*An. barberellus, An. cinctus, An. coluzzii, An. coustani, An. funestus s.l., An. gambiae s.s.,* *An. hancocki, An. hargreavesi, An. melas, An. nili, An. obscurus, An. paludis, An. pretoriensis,* *An. smithii, An. squamosus, An. ziemanni*
**Madagascar**	**1169** (1902–2014) [96]	**829** [72]	*An. arabiensis, An. brunnipes, An. coustani, An. cydippis, An. flavicosta, An. funestus s.s.,* *An. fuscicolor, An. gambiae s.s., An. grassei, An. grenieri, An. griveaudi, An. lacani, An. maculipalpis,* *An. mascarensis, An. merus, An. milloti, An. notleyi, An. pauliani, An. pharoensis, An. pretoriensis, An. radama, An. ranci,* *An. roubaudi, An. rufipes, An. squamosus, An. tenebrosus*
**Malawi**	**220** (1900–2015) [133]	**19** [9]	*An. arabiensis, An. cinereus, An. coustani, An. demeilloni, An. distinctus, An. funestus s.s.,* *An. gambiae s.s., An. longipalpis, An. maculipalpis, An. marshallii, An. parensis, An. pharoensis,* *An. pretoriensis, An. rhodesiensis, An. rivulorum, An. rufipes, An. seydeli, An. squamosus,* *An. tenebrosus, An. ziemanni*
**Mali**	**430** (1906–2014) [82]	**163** [14]	*An. arabiensis, An. brohieri, An. brunnipes, An. coluzzii, An. coustani, An. domicolus, An. flavicosta, An. funestus s.l.,* *An. gambiae s.s., An. hancocki, An. leesoni, An. longipalpis, An. maculipalpis, An. nili, An. obscurus, An. paludis, An. pharoensis,* *An. pretoriensis, An. rhodesiensis, An. rivulorum,* *An. rufipes, An. sergentii, An. squamosus, An. wellcomei, An. ziemanni*
**Mauritania**	**160** (1908–2013) [28]	**107** [17]	*An. arabiensis, An. coluzzii, An. coustani, An. dthali, An. demeilloni, An. domicolus,* *An. freetownensis, An. funestus s.l., An. hancocki, An. melas, An. pharoensis, An. pretoriensis,* *An. rhodesiensis, An. rufipes, An. squamosus, An. ziemanni*
**Mayotte**	**15** (1952–2011) [1]	**4** [0]	*An. coustani, An. funestus s.l., An. gambiae s.s., An. maculipalpis, An. mascarensis, An. pretoriensis*
**Mozambique**	**191** (1900–2014) [49]	**72** [10]	*An arabiensis, An. brunnipes, An. cinereus, An. confusus, An. coustani, An. cydippis, An. demeilloni, An. funestus s.s.,* *An. gambiae s.s., An. leesoni, An. letabensis, An. listeri, An. longipalpis,* *An. maculipalpis, An. marshallii, An. merus, An. mousinhoi, An. natalensis, An. nili, An. paludis,* *An. pharoensis, An. pretoriensis, An. quadriannulatus, An. rhodesiensis, An. rivulorum, An. rufipes, An. seydeli, An. squamosus,* *An. tenebrosus, An. theileri, An. wellcomei, An. ziemanni*
**Namibia**	**57** (1934–2004) [0]	**23** [0]	*An. arabiensis, An. cinereus, An. coustani, An. demeilloni, An. distinctus, An. fontinalis,* *An. funestus s.s., An. gambiae s.s., An. listeri, An. maculipalpis, An. marshallii, An. moucheti,* *An. namibiensis, An. nili, An. pharoensis, An. pretoriensis, An. quadriannulatus, An. raurinus,* *An. rhodesiensis, An. rivulorum, An. rufipes, An. squamosus, An. vaneedeni, An. ziemanni*
**Niger**	**112** (1950–2010) [18]	**53** [8]	*An. arabiensis, An. coluzzii, An. coustani, An. dthali, An. funestus s.l., An. gambiae s.s.,* *An. maculipalpis, An. multicolor, An. nili, An. pharoensis, An. pretoriensis, An. rhodesiensis,* *An. rhodesiensis rupicolus, An. rivulorum, An. rufipes, An. salbaii, An. squamosus, An. wellcomei,* *An. ziemanni*
**Nigeria**	**391** (1909–2016) [150]	**118** [45]	*An. arabiensis, An. barbarellus, An. brohieri, An. brucei, An. brunnipes, An. cinctus, An. coluzzii,* *An. coustani, An. cristipalpis, An. domicolus, An. flavicosta, An. freetownensis, An. funestus s.s.,* *An. gambiae s.s., An. hancocki, An. hargreavesi, An. implexus, An. jebudensis, An. leesoni,* *An. maculipalpis, An. marshallii, An. melas, An. moucheti nigeriensis, An. nili, An. obscurus,* *An. paludis, An. pharoensis, An. pretoriensis, An. rhodesiensis, An. rivulorum, An. rufipes,* *An. smithii, An. squamosus, An. theileri, An. watsoni, An. wellcomei, An. ziemanni*
**Rwanda**	**78** (1933–2014) [40]	**37** [5]	*An. arabiensis, An. ardensis An. christyi, An coustani, An. demeilloni, An. dureni, An. funestus s.l.,* *An. gambiae s.s., An. garnhami, An. implexus, An. maculipalpis, An. marshallii, An. moucheti,* *An. natalensis, An. nili, An. paludis, An. pharoensis, An. pretoriensis, An. squamosus,* *An. tenebrosus, An. ziemanni*
**Sao Tome &** **Principe**	**57** (1945–2004) [0]	**14** [0]	*An. coluzzii, An. coustani, An. funestus s.l., An. melas, An. paludis, An. pharoensis*
**Senegal**	**513** (1902–2014) [292]	**254** [147]	*An. arabiensis, An. brohieri, An. brunnipes, An. coluzzii, An. coustani, An. domicolus, An. flavicosta, An. freetownensis,* *An. funestus s.s., An. gambiae s.s., An. hancocki, An. maculipalpis, An. melas, An. nili, An. paludis, An. pharoensis, An. pretoriensis,* *An. rufipes, An. squamosus, An. wellcomei,* *An. ziemanni*
**Sierra Leone**	**186** (1898–2012) [25]	**39** [2]	*An. barberellus, An. brohieri, An. brunnipes, An. cinctus, An. coluzzii, An. coustani, An. domicolus, An. flavicosta,* *An. freetownensis, An. funestus s.l., An. gambiae s.s., An. hancocki, An. hargreavesi,* *An. marshallii, An. melas, An. moucheti, An. nili, An. obscurus, An. paludis, An. pharoensis,* *An. quadriannulatus, An. rhodesiensis, An. rufipes, An. smithii, An. somalicus, An. squamosus,* *An. tenebrosus, An. theileri, An. ziemanni*
**Somalia**	**413** (1935–2014) [164]	**25** [6]	*An. arabiensis, An. azaniae, An. cinereus, An. coluzzii, An. coustani, An. culicifascies, An. demeilloni, An. daudi, An. dthali,* *An. funestus s.l., An. merus, An. nili, An. paludis, An. pharoensis, An. pretoriensis, An. rhodesiensis, An. salbaii, An. sergentii* *macmahoni, An. somalicus, An. squamosus, An. turkhudi*
**South Africa**	**184** (1903–2016) [18]	**114** [16]	*An. arabiensis, An. ardensis, An. argenteolobatus, An. azevedoi, An. cameroni, An. carteri,* *An. cinctus, An. cinereus, An. confusus, An. crypticus, An. cydippis, An. demeilloni, An. funestus s.s.,* *An. garnhami, An. hughi, An. implexus, An. kosiensis, An. leesoni, An. letabensis, An. listeri,* *An. longipalpis, An. maculipalpis, An. marshallii, An. merus, An. mousinhoi, An. natalensis, An. nili, An. parensis, An. pharoensis,* *An. pretoriensis, An. quadriannulatus, An. rhodesiensis, An. rivulorum, An. ruarinus, An. rufipes, An. squamosus, An. tenebrosus,* *An. turkhudi, An. vaneedeni, An. vernus, An. ziemanni*
**South Sudan**	**115** (1903–2010) [5]	**55** [2]	*An. arabiensis, An. brohieri, An. coustani, An. demeilloni, An. flavicosta, An funestus s.l.,* *An. garnhami, An. implexus, An. leesoni, An. longipalpis, An. maculipalpis, An. marshallii,* *An. moucheti, An. nili, An. obscurus, An. paludis, An. pharoensis, An. pretoriensis, An. rhodesiensis, An. rivulorum, An. rufipes,* *An. sergentii, An. squamosus, An. symesi, An. wellcomei, An. ziemanni*
**Sudan**	**459** (1903–2014) [171]	**165** [42]	*An. arabiensis, An. cinereus, An. coustani, An. dthali, An. funestus s.s., An. leesoni, An. maculipalpis,* *An. marshallii, An. multicolor, An. nili, An. paludis, An. pharoensis, An. pretoriensis, An. rhodesiensis,* *An. rhodesiensis rupicolus, An. rivulorum, An. rufipes, An. sergentii, An. squamosus, An. turkhudi, An. wellcomei, An. ziemanni*
**Swaziland**	**14** (1960–1992) [0]	**6** [0]	*An. arabiensis, An. coustani, An. caliginosus, An. funestus s.l., An. maculipalpis, An. marshallii,* *An. merus, An. nili, An. pretoriensis, An. quadriannulatus, An. rivulorum, An. rufipes, An. squamosus*
**Tanzania** **(Mainland)**	**524** (1900–2014) [193]	**80** [32]	*An. arabiensis, An. ardensis, An. argenteoIobatus, An. brunnipes, An. cinereus, An. confusus,* *An. coustani, An. christyi, An. cydippis, An. demeilloni, An. distinctus, An. funestus s.s.,* *An. gambiae s.s., An. garnhami, An. gibbonsi, An. implexus, An. keniensis, An. kingi, An. leesoni,* *An. longipalpis, An. lovettae, An. machardyi, An. maculipalpis, An. marshallii, An. merus,* *An. natalensis, An. nili, An. njombiensis, An. paludis, An. parensis, An. pharoensis, An. pretoriensis, An. quadriannulatus,* *An. rhodesiensis, An. rivulorum, An. rufipes, An. schwetzi, An. seydeli,* *An. squamosus, An. swahilicus, An. tenebrosus, An. theileri, An. vaneedeni, An. wellcomei,* *An. wilsoni, An. ziemanni*
**Togo**	**106** (1902–2013) [3]	**22** [1]	*An. arabiensis, An. brunnipes, An. coluzzii, An. coustani, An. flavicosta, An. funestus s.l.,* *An. gambiae s.s., An. hargreavesi, An. maculipalpis, An. melas, An. nili, An. obscurus, An. paludis,* *An. pharoensis, An. pretoriensis, An. rhodesiensis, An. rivulorum, An. rufipes, An. squamosus,* *An. wellcomei, An. ziemanni*
**Uganda**	**383** (1907–2013) [174]	**158** [40]	*An. arabiensis, An. ardensis, An. bervoetsi, An. brohieri, An. bwambae, An. christyi, An. cinereus,* *An. coustani, An. cydippis, An. demeilloni, An. domicolus, An. funestus s.s., An. gambiae s.s.,* *An. garnhami, An. gibbinsi, An. hancocki, An. hargreavesi, An. harperi, An. implexus, An. keniensis, An. kingi, An. leesoni,* *An. longipalpis, An. maculipalpis, An. marshallii, An. moucheti, An. natalensis, An. nili, An. obscurus, An. paludis, An. parensis,* *An. pharoensis, An. pretoriensis, An. rhodesiensis, An. rivulorum, An. rufipes, An. squamosus, An. symesi, An. tenebrosus,* *An. vinckei, An. wellcomei, An. ziemanni*
**Zambia**	**155** (1932–2015) [127]	**29** [16]	*An. arabiensis, An. argenteolobatus, An. brunnipes, An. coluzzii, An. coustani, An. demeilloni,* *An. distinctus, An. domicolus, An. funestus s.s., An. gambiae s.s., An. implexus, An. leesoni,* *An. longipalpis, An. maculipalpis, An. marshallii, An. nili, An. parensis, An. pharoensis,* *An. pretoriensis, An. quadriannulatus, An. rhodesiensis, An. rivulorum, An. rufipes, An. schwetzi,* *An. seydeli, An. squamosus, An. theileri, An. vaneedeni, An. walravensi, An. wellcomei, An. ziemanni*
**Zanzibar**	**91** (1913–2014) [22]	**4** [1]	*An. arabiensis, An. aruni, An. coustani, An. funestus s.l., An. leesoni, An. longipalpis, An. maculipalpis, An. marshallii, An. merus,* *An. obscurus, An. paludis, An. parensis, An. pretoriensis,* *An. quadriannulatus, An. rivulorum, An. squamosus, An. swahilicus, An. tenebrosus, An. wellcomei, An. ziemanni*
**Zimbabwe**	**109** (1901–2014) [29]	**38** [7]	*An. arabiensis, An. ardensis, An. argenteolobatus, An. brunnipes, An. carteri, An. cinereus,* *An. coluzzii, An. confusus, An. coustani, An. cydippis, An. demeilloni, An. domicolus, An. funestus s.s.,* *An. fuscivenosus, An. gambiae s.s., An. garnhami, An. leesoni, An. listeri, An. longipalpis,* *An. maculipalpis, An. marshallii, An. merus, An. mousinhoi, An. natalensis, An. nili, An. parensis,* *An. pharoensis, An. pretoriensis, An. quadriannulatus, An. rhodesiensis, An. rivulorum, An. ruarinus, An. rufipes, An. schwetzi,* *An. seydeli, An. squamosus, An. tenebrosus, An. theileri, An. vaneedeni,* *An. walravensi, An. wellcomei, An. ziemanni*

**Figure 2.**
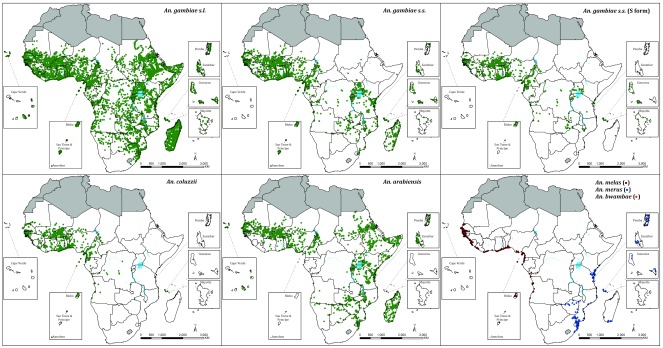
Distribution of sampled sites reporting presence of *Anopheles gambiae* complex (11,494), and sibling species, regarded as dominant vectors of malaria within their ecological range. *An. gambiae* s.s (3,988 locations) is shown separately to allow for reporting that did not distinguish, for example, between *An. coluzzii*/M forms and *An. gambiae* S forms. *An. gambiae s.s* (S form) 1,574 locations, *An. coluzzii* 1,331 locations, *An. arabiensis* = 3,635 locations, *An. melas* 538 locations, *An. merus* 253 locations and *An. bwambae* 26 locations.

**Figure 3.**
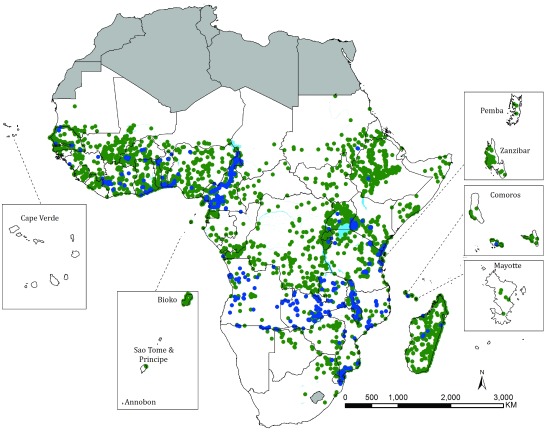
Distribution of 5,052 sampled locations reporting the presence of *Anopheles funestus* s.l. (green) or where survey reports specified *An. funestus* s.s. (Blue) (n=727).

### Dominant vectors

**Figure 4.**
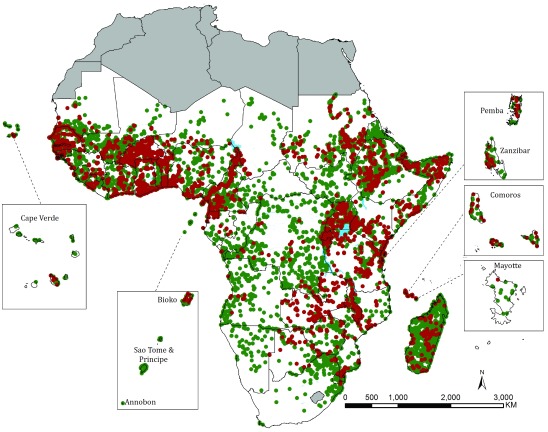
Distribution of sampled locations reporting the presence of dominant vectors *Anopheles nili* s.l., *An. moucheti* and *An. mascarensis*. ( **A**) *An. nili* s.l. (n = 822 locations); ( **B**) *An. moucheti* s.l. (n = 499 locations), the two sites in Namibia were noted as unusual for this far south ( De Meillon, 1951) and ( **C**) *An. mascarensis* (n = 483 locations).

Within the Gambiae complex, *An. arabiensis* has a more extensive range ( Figure 2), including the more arid areas south of the Sahara and the Horn of Africa. Conversely, *An. gambiae s.s.* and *An. coluzzii* have been described more frequently in West Africa compared to Central and East Africa. The saltwater breading *An. melas* has a restricted range along the West African coast; however the ranges of *An. merus* in East and Southern Africa show an extended, inland geographic range ( Figure 2) ( Bushrod, 1981; Coetzee *et al.*, 1993; Cuamba & Mendis, 2009; Kigadye *et al.*, 2010). *An. funestus s.s.,* the dominant malaria vector within its group, has only recently been described uniquely through advances in molecular techniques, its distribution being within the range of more ubiquitous data on *An. funestus s.l.* ( Figure 3). *An. moucheti s.l.* is located principally in central Africa, but described further west across Nigeria and recorded as far west as Sierra Leone and Guinea ( Figure 4). *An. nili s.l.* has a more extended range ( Figure 4) covering areas occupied by other dominant vectors. While *An. mascarensis*, also a dominant vector within its range, is constrained to Madagascar, Comoros and Mayotte ( Figure 4).

### Secondary vectors

**Figure 5.**
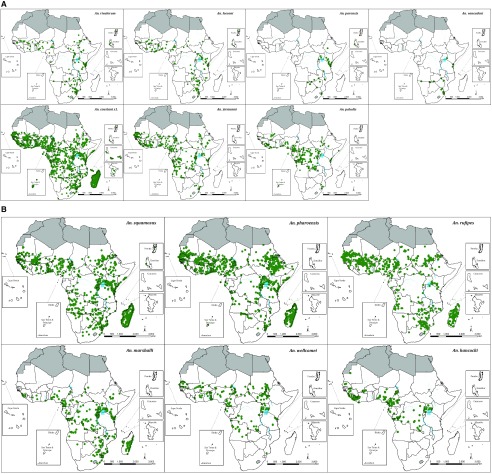
Distribution of sampled locations of potential secondary malaria vectors ( Table 1). ( **A**) *Anopheles funestus* group: *An. rivulorum* (n = 244), *An. leesoni* (n = 187), *An. parensis* (n = 41) and *An. vaneedeni* (n = 34); the *An. coustani* group (n = 2,689) and where specified sibling species *An. ziemanni* (n = 662) and *An. paludis* (n = 375). ( **B**) *An. squamosus* (n= 1,294), *An. pharoensis* (n = 1,889), *An. rufipes* (n = 998), *An. marshallii* (n = 442), *An. wellcomei* (n = 272) and *An. hancocki* (n = 297).

Fewer site locations reported possible secondary vectors compared to reporting of dominant vectors. We identified survey reports of potential secondary vectors ( Table 1) at 4,442 site locations sampled since 1901, and only 1,106 site locations where the sampling date included 2005–2016. Among the possible secondary vectors of the *An. funestus* group both *An. rivulorum* and *An. leesoni* showed an extensive range across SSA ( Figure 5A), there were fewer reports of *An. parensis* and *An. vaneedeni* presence; however these were largely at sites located in Eastern and Southern Africa ( Figure 5A). Reports of the presence of members of the *An. coustani* group were ubiquitous and extensive in their range ( Figure 5A), while sibling species of this group, *An. ziemanni* and *An. paludis*, were more frequently reported within a constrained range across the central belt of Africa ( Figure 5A).

*An. rufipes*, *An. pharoensis*, *An. squamosus* and *An. marshallii* all have a cosmopolitan range across Africa ( Figure 5B). *An. hancocki* and *An. wellcomei* have been largely reported from the central African belt ( Figure 5B).

## Discussion

The geo-coded inventory of anopheline species in SSA covers over 13,000 locations and represents the most spatially comprehensive description of dominant and secondary malaria vectors in Africa to-date. Of the sites sampled since the turn of the last century, 4,494 (33.7%) have been sampled at least once since 2005 ( Figure 1). The use of historical archive and unpublished material has been fundamental in expanding our understanding of the spatial ranges of primary and secondary malaria vectors in Africa. Our assembly process highlights the significance of ensuring unpublished materials are sourced at country levels. A wealth of unpublished information existed 50 years ago and this is equally true today. We have not visited every country in Africa nor visited every national malaria control programme and university archive on the continent. The data on anopheline vectors could be improved substantially at country levels.

We have not attempted to model the likely presence of dominant or secondary vectors where currently no data exist. This has been attempted by others ( Foley *et al.*, 2010; Levine *et al.*, 2004; Lunde *et al.*, 2013; Moffett *et al.*, 2007; Sinka *et al.*, 2010; Wiebe *et al.*, 2017) and used to project the likely ranges of dominant vectors under different climate ( Drake & Beier, 2014) and intervention ( Sinka *et al.*, 2016) scenarios. It is our belief that modelling cannot replace actual field survey data. In addition, we cannot reliably document the temporal changes in vector species compositions. The precision in species identification has changed with time, making it hard to compare pre-1990 dominant species complex composition with current compositions, and relative abundance is often not reported. Such time-series analysis is better described at small, site specific locations of repeat, standardised sampling rather than from large summary database repositories as presented here. 

Recent modelling exercises to predict niches, using climate and ecological predictors of vector species presence ( Foley *et al.*, 2010; Levine *et al.*, 2004; Lunde *et al.*, 2013; Moffett *et al.*, 2007; Sinka *et al.*, 2010; Wiebe *et al.*, 2017), largely confirm the natural geographic ranges described during early cartographies of species distributions in Africa ( Gillies & de Meillon, 1968). Likewise, we show the constrained, and sympatric, ranges of *An. gambiae* and *An. funestus* groups across the west, central and southern African belts where these highly efficient vector groups continue to contribute to some of the highest *P. falciparum* transmission rates in Africa ( Noor *et al.*, 2014). This ecological range for the most significant of malaria vectors is unlikely to change dramatically with more data or more elaborate modelling. However, far less is known empirically about the possible secondary vectors of malaria in Africa ( Table 1). 

Secondary vectors of malaria might become increasingly important as indoor-centric vector control efforts change the landscape of dominant vector compositions, resulting in residual transmission being maintained by outdoor biting and resting vectors ( Afrane *et al.*, 2016). The description of the complete range of anophelines at sampled locations is often incomplete, largely because sampling strategies focus on dominant indoor biting and resting vectors. Therefore, in the absence of outdoor adult mosquito sampling, possible secondary vectors are not documented ( Stevenson & Norris, 2017). It is important to note that these predominantly zoophilic mosquitoes do at times feed on humans and can be found resting indoors. However, more information is required beyond simply defining their ecological range to be able to interpret the importance of these potential vectors of residual malaria transmission in Africa. Abundance is a key feature of a vector’s ability to transmit malaria, and often these data are not readily available from reports of secondary vectors. Some anophelines, however, have other roles in public health, notably their ability to transmit other parasites and viral infections and contributing to the transmission of: filariasis ( *An. pharoensis*) ( Gillies & de Meillon, 1968), Rift Valley fever ( *An. coustani, An. squamosus*) ( Tantely *et al.*, 2013) ( *An. pharoensis*, *An. rufipes*, *An. coustani*) Zika virus ( *An coustani s.s.*, *An paludis*) and Chikungunya virus ( *An. rufipes*, *An. coustani*) ( Tantely *et al.*, 2016).

The most recent geo-coded inventory of anophelines in Africa focused only on dominant vectors, published and contemporary data sources ( Wiebe *et al.*, 2017). We have used unpublished national reports dating back to the period before the GMEP, which have provided a rich source of additional information related to dominant and secondary malaria vectors, spanning over 100 years. This geo-coded repository of data is provided on Harvard Dataverse ( Snow, 2017), and original source materials have been provided to the Global Malaria Programme of the WHO. These data are therefore available to every national malaria agency responsible for the future control or elimination of malaria across SSA. In addition, the data are also available to national academic counterparts to malaria programmes in SSA interested in vector species niche mapping. We imagine that a first step is that the maps of sampled presence of dominant vectors, their sibling species and potential secondary vectors will be used to highlight where information within national borders is currently absent. Linking mapped species distributions to information on insecticide resistance provides a layered, information platform to manage insecticide use (IRBase; Coleman *et al.*, 2017).

Using information to plan an effective control programme is crucial, this was recognized over 50 years ago in SSA, but conspicuous by its absence at the launch of the recent Roll Back Malaria initiative. As malaria programmes need to become more selective, nuanced and focused in the application of interventions, data platforms are an essential part of the preparation stage. There is a growth in sub-national targeting of resources to meet epidemiological needs, driven largely by variations in empirical data on malaria infection prevalence or routine clinical data. We would encourage countries to build their knowledge base on malaria vector species compositions as part of a broader epidemiological profile within their national borders.

## Data availability

The data referenced by this article are under copyright with the following copyright statement: Copyright: © 2017 Kyalo D et al.

The Vectors Database, which includes all the data that support the findings of this study, are available from the KEMRI Wellcome Trust Research Programme’s Population Health Dataverse, http://dx.doi.org/10.7910/DVN/NQ6CUN ( Snow, 2017), under a CC-BY 4.0 license.
