# Supplementary material for: A geo-coded inventory of anophelines in the Afrotropical Region south of the Sahara: 1898-2016
Source: Wellcome Open Res. 2017 Jul 26;2:57. [Version 1] doi: 10.12688/wellcomeopenres.12187.1 (PMC5558104; doi:10.12688/wellcomeopenres.12187.1)
Supplement: Peer review history

# Peer review: Referee response for version 1

**DOI:** 10.21956/wellcomeopenres.13191.r24760

**Published:** 2017-08-30

**Authors:** Rousseau Djouaka

**Affiliations:** 1 AgroEcoHealth Platform, International Institute of Tropical Agriculture (IITA), Cotonou, Benin

This submitted manuscript is very relevant for malariologists including policy/decision makers working on malaria vector control. The authors have generated a very impressive set of data from 1898 to 2016 to map and report Anopheles distribution in Africa. This review is very unique in the sense that: (i) it builds on data of more than 118 years old , (ii) it includes both published and unpublished references collected from several libraries and institution archives, (iii) could serve as strong starting  point for other research works such as: (a) Mapping and identifying some key historical events which occurred on the dynamics/distribution of malaria vectors in Africa over the past 100 years, (b) generate information on climate changes/variations (when these data are coupled with climatic parameters) and analyse how changing climate conditions have affected the distribution of malaria vectors over the past 100 years in Africa.

 As complementary information, I will make 2 suggestions:

- Authors should be more precise on the duration it took to generate such volume of information (…See Abstract Results:  “It took several years … How many years?”
- Authors should clearly describe (under the methodology section) the selection/rejection criteria used for unpublished data and how these data were cleaned before been incorporated among the set of published data.

I have read this submission. I believe that I have an appropriate level of expertise to confirm that it is of an acceptable scientific standard.

# Peer review: Referee response for version 1

**DOI:** 10.21956/wellcomeopenres.13191.r24527

**Published:** 2017-08-11

**Authors:** Michael J. Bangs

**Affiliations:** 1 International SOS Public Health & Malaria Control, Kuala Kencana, Indonesia

Kyalo et al.  provide an exceptional paper and excellent summarization (detailed, yet concise) with important historical references to produce a timely resource covering approximately 118 years of observations on anopheline mosquitoes and their respective distributions in sub-Saharan Africa.  A number of painstaking years in production, the scope of the coverage is impressive.  Data covers not only published accounts but also the partially hidden ‘grey’ literature and miscellaneous reports lurking in the dusty recesses of government archives and elsewhere.  As the authors point out, what is presented as inventory is actual recorded collections of specimens, not just predicted distributions (albeit useful in its own right).  Both approaches have merit and inherent limitations as to what the data can and cannot provide or be extrapolated upon.

 Although the systematics of the anopheline mosquitoes has resulted in revised taxonomy and nomenclature over the many decades (precision in species identification, expanding groups and complexes, refined phylogenetic relationships, for example), one overriding theme to these assembled works has been a greater understanding of the mosquitoes’ complex role in malaria transmission in specific areas.  Where competent vectors exist (primary or secondary), perennial or seasonal, the specter of transmission risk persists. 

 The compiled inventory is a prime example of Science as a series of building blocks, with history serving as its foundation (with the occasional evolving paradigm shift).  Never lose sight of the past – it can be invaluable no matter the age.  Historical references provide both a temporal-spatial ‘baseline’ and confirmation of vector presence that can too easily become ‘lost’, thereby denying a larger audience of invaluable data on specific locations that might not be revisited for a long time to come.  Moreover, this article serves as a template to emulate in other malaria endemic regions of the world – for those so daring. 

 This publication provides a solid foundation from which to continue to add ‘points’ to the African map as older information continues to surface and new data is gathered.  Despite the monumental effort to assemble and display vector distributions, the authors acknowledge there remain documents yet to recover. To point, what is not clear in the presentation is how interested individuals and institutions might be able to contribute additional entries to the vector databases (Harvard Dataverse, KEMRI Vectors Database) from information still ‘hidden’ and yet to be captured.  

I have read this submission. I believe that I have an appropriate level of expertise to confirm that it is of an acceptable scientific standard.

# Peer review: Referee response for version 1

**DOI:** 10.21956/wellcomeopenres.13191.r24525

**Published:** 2017-08-07

**Authors:** Josiane Etang

**Affiliations:** 1 Organisation for Coordination in the Fight Against Endemic Diseases in Central Africa (OCEAC), Yaoundé, Cameroon

The subject is of relevance to malaria entomology and will be of significant interest to those in the field of malaria control or elimination in Sub-Saharan Africa. The team of authors has a significant track record in this area, having previously carried out major surveys of malaria vectors in Africa. The paper provides an up-to-date catalogue of recorded anopheline species for the Afrotropical region, based on data extracted from peer-reviewed published sources and unpublished research reports, which allow to gather a wide-range of information on primary and secondary malaria vectors in Africa.

 However, following are two issues to address:

1. It appears that important sources of information have been national inventories developed by entomologists working in Africa, which sounds very interesting. Furthermore, the use of historical archive and unpublished material has been fundamental in expanding the understanding of the spatial ranges of primary and secondary malaria vectors. However, the authors have not provided a detailed description of data sources to highlight the contribution of unpublished material in the inventory of anopheles that is being published. It would be interesting to build a table or a figure on breakdown of published and unpublished data.
2. The authors made a huge effort to gather recent data including those from islands; recent data on malaria vectors in the island of Manoka in Cameroon (Mbida *et al*, 2017, Etang *et al*. 2017…) may also be included.

I have read this submission. I believe that I have an appropriate level of expertise to confirm that it is of an acceptable scientific standard.
